# Emerging directions in the study of the environmental determinants of mental health: commentary on the MINDMAP Project

**DOI:** 10.1136/jech-2021-216713

**Published:** 2021-04-07

**Authors:** Peter James, Ichiro Kawachi

**Affiliations:** 1 Department of Population Medicine, Harvard Medical School and Harvard Pilgrim Health Care Institute, Boston, Massachusetts, USA; 2 Environmental Health, Harvard University T H Chan School of Public Health, Boston, Massachusetts, USA; 3 Social and Behavioral Sciences, Harvard University T H Chan School of Public Health, Boston, Massachusetts, USA

**Keywords:** mental health, environmental epidemiology, ageing

The study of environmental determinants of health is at a crossroads. Harmonised health data across cohorts followed over decades, novel technologies to gather information on health behaviours and location data, and high-resolution spatial data on environmental factors have made it possible for researchers to unearth insights and relationships never before possible. This special issue of *Journal of Epidemiology and Community Health* brings findings from collaborators in the MINDMAP Project, an ambitious effort to examine the environmental determinants of mental health and well-being in older populations across Europe and Canada. The investigators involved in these studies have developed multiple high-resolution spatial datasets to examine a broad range of environmental factors, including area-level socioeconomic measures, crime, the built environment, green spaces and noise. In addition, the MINDMAP collaboration enables validated and harmonised measures of mental health and well-being, including loneliness, depressive symptoms, antidepressant use, anxiety, affect and mental distress. But the true strength of the MINDMAP collaboration is the potential for innovation by applying diverse study designs, ranging from mobile health approaches to agent-based modelling, to answer questions about how environmental factors drive healthy ageing. The findings presented unearth insights into potential environmental drivers of healthy ageing.

## Overview of MINDMAP

Wey *et al* provide an overview of the MINDMAP Project, which used longitudinal data from six cohort studies located in Eastern and Western Europe, as well as Canada, that comprised a total of 220 621 participants. Baseline years of these studies ranged from 1984 to 2012, with up to seven repeated data collection periods. Looking across these studies, the investigators harmonised data on 1848 environmental exposures and 993 individual-level determinants and health outcomes. The domains covered by these rich harmonised data include physical environments, sociodemographic factors, health behaviours, disease status, medication use, cognitive functioning, psychological assessments and social networks. The resulting harmonised multinational dataset was transparently documented and stored on a central MINDMAP server for analysis.

Introducing the complexity of ageing and well-being, Dapp *et al* capitalised on longitudinal MINDMAP data to examine the dynamics between depression, frailty and disability within an older cohort in Hamburg, Germany. The authors observed that depression increased the risk of subsequent frailty, and that frailty increased the risk of subsequent depression. Interestingly, the investigators saw that while depression increased the risk of subsequent disability, disability was not associated with higher risk of subsequent depression. Dapp *et al* provide novel perspectives into the processes between ageing, mental health and disability, and offer suggestions for increasing screening for depressed mood and functional decline to produce timely and targeted interventions.

## The importance of theory

Theory may sharpen predictions about how urban environments influence mental well-being in old age. There is a lack of consensus on even basic descriptive questions such as whether the prevalence of depressive symptoms rises with advancing age, and therefore inconsistencies in the empirical literature can only be reconciled and understood with the aid of good theory. In particular, multilevel studies of neighbourhood environments and mental health are often missing a third, higher, level of organisation, that is, the *societal context* in which people live their lives. This is only made possible by careful cross-national comparisons of harmonised data.

To give a detailed example of what can be learnt from cross-national comparisons, a recent study contrasted suicide rates in Japan and South Korea, two neighbouring countries which share many superficial similarities (eg, rapid population ageing and high suicide rates overall), yet starkly different suicide rates at older ages.^1^ Applying age–period–cohort analysis of suicide trends between 1986 and 2015, Kino *et al* showed that there is a sharp increase in suicide around retirement age in Korea, but not in Japan (an age effect). Furthermore, there was a dramatic temporal increase in suicide during the three decades of observation in Korea (a period effect) whereas rates were relatively stable in Japan. Lastly, the post-World War II generation in Japan had lower rates of suicide compared with generations born either before 1916 or after 1961 (birth cohort effect), whereas the suicide rate increased linearly with each generation in Korea. Japan provides a strong social safety net for the generation who contributed to the post-war period of economic expansion, while high suicide rates in Korea reflect the simultaneous decline of intergenerational care provision combined with inadequate social security in post-retirement. Thus, although Japan and Korea share high *overall* suicide rates, careful cross-national comparative analysis points to divergent social policies as the basis for the stark differences in suicide at older ages. This example highlights how difficult it is to generalise about population variability in mental health without an adequate understanding of the broader social context (particularly the social policy context) in which older adults lead their lives. Urban contexts are embedded within upstream social contexts. Hence, whether a research study conducted in country X confirmed/disconfirmed the findings of another study conducted in country Y is hard to interpret without considering the ‘missing level’ above urban neighbourhoods.

Turning to the MINDMAP Project, Tarkiainen *et al* argue that the association between neighbourhood characteristics and mental health at older ages has produced inconsistent findings, possibly due to heterogeneity in the measurement of mental health outcomes, neighbourhood characteristics and confounders. In their cross-national comparative study, which harmonised measures of exposures, outcomes and confounders across three countries—Finland, Sweden and Italy—the authors found that dense and mixed urban structure was associated with higher antidepressant use at older ages in Stockholm and in Finland, but not in Italy. In other words, their study buttresses the idea that there is something more going on than measurement and study design issues, and heterogeneity of treatment effects *might be expected* depending on the social context. Tarkiainen *et al* speculate that their mixed finding might be explained by differences in family solidarity (a cultural characteristic) between the countries, viz. Italy is characterised by strong family responsibility for older people while contact with elderly parents may be looser in the Nordic countries (Indeed, the frequency of intergenerational contact has been put forward as one of the reasons why Italy suffered one of the worst COVID-19 outbreaks in Europe.^2^). Future studies might attempt to incorporate these measures of social context into analysis to better understand the mechanisms at play.

## Improving exposure assessment

Exposure assessment is at the crux of research on environmental drivers of health. Accurate exposure assessment that reflects personal exposure during a relevant time window allows for more precise estimation of the relationship between an environmental factor and healthy ageing. Conversely, non-differential measurement error is likely to bias results towards the null.^3^ Therefore, if the exposures estimated across the studies in this special issue contain non-differential error, it is possible that this error accounts for the majority of null findings.

While evidence is growing that environmental factors may drive mental health and well-being as we age, limitations in exposure assessment are the largest barriers to advancing the field. Poorly measured exposure data do not allow us to determine aetiologically relevant exposures in a way that is actionable by individuals or communities. Coarse exposure assessment limits statements about causal inference and provides little information on potential interventions for policymakers.^4 5^

This lack of consistency in defining exposures could be at play in the study by Tarkiainen *et al*, where the authors observed inconsistent associations for antidepressant use by levels of urbanicity, land use mix, and population density across areas of Sweden, Finland and Italy. The definition of dense urban structure may differ greatly in Sweden and Finland compared with Italy. Are dense neighbourhoods monolithic apartment complexes or mixed-use vibrant communities? While both scenarios would constitute high density, the lack of a well-defined exposure makes it difficult to discern what the true exposure is that might drive antidepressant use. In addition, urbanicity is defined as ‘proportion of continuous urban fabric’. How would one design a randomised trial to experimentally expose someone to ‘urbanicity’? And, assuming urbanicity does cause antidepressant use, how would researchers advise policymakers on how to change urbanicity? Do we remove pavement? Knock down buildings? Plant trees? Broadly defined exposures create confusion in understanding exactly what causal question we are asking.

Similarly, other studies used non-specific measures of the built environment in analyses, including Ruiz *et al*, Sund *et al* and Noordzij *et al*. Noordzij *et al* define exposure to green space based on the distance between a participant’s residential address and the nearest green space using data from the Urban Atlas dataset, which contains comparable land use and land cover data across Europe. The use of a harmonised green space metric allows for pooling of the data across all four cohorts; however, the downside is that we have no information on the specific type of green space involved. Are grassy meadows comparable with wooded forests? Are urban parks comparable with suburban parks? The combination of these dissimilar green spaces, where some may positively influence depressive symptoms and others might not, contributes to exposure misclassification. The authors in Sund *et al* mention that urban areas provide an urban penalty by increasing exposure to air pollution, noise or violence, or conversely, may provide an urban advantage by providing higher access to cultural activities or social networks. Future MINDMAP studies should measure and estimate the effects of these specific factors on health.

Timmermans *et al* conducted an analysis on land use and loneliness in older adults from a cross-sectional analysis of two Dutch cohorts. In the time of COVID-19 and increased social distancing, understanding environmental drivers of loneliness is all the more important. The authors find some suggestion that participants living in areas with higher land use mix had lower levels of loneliness, although this finding was not statistically significant. The authors proffer that land use mix could reflect ‘the availability of various destinations and neighbourhood resources in the local living environment’; however, land use mix could also be correlated with other factors, such as access to transit, access to green spaces or even something as simple as street benches, which encourage social interaction. Future research could engage multiexposure models to isolate which specific factor appears to have the greatest impact on loneliness.

Li *et al* evaluated whether a noise mitigation policy in Amsterdam led to an improvement in mental health. There are theoretical and empirical reasons why noise can affect residents’ mental health (not the least through sleep disruption). From an exposure assessment perspective, one of the things that researchers seldom bother to assess is how do the residents perceive noise. When people appraise the noise as unpredictable, beyond their control and not to their benefit, the mental health impacts are much worse. If, however, there are more positive appraisals (eg, residents have been told that the noise will last for a specified duration of time and is associated with some community benefit—for example, the construction of an attractive neighbourhood amenity—the mental health impacts will be less). Self-reported data on noise perceptions, as well as control over noise, would be a worthwhile addition to the MINDMAP Project.

## Technological advances to address gaps

Recent technological advances have provided researchers with tools that can fill many research gaps outlined above. We have new tools to estimate high-resolution metrics of mobility, human behaviour and psychological processes that occur within a day. Fernandes *et al* describe the development of a study that incorporates multiple tools for innovative perspectives on these factors. Their research protocol combines global positioning systems and accelerometer data, proximity detection to assess whether household members are close to each other for objective measures of social interactions, ecological momentary assessment prompts up to eight times per day to track momentary mood and stress and environmental perceptions, and electrodermal activity for the potential objective prediction of stress. These technologies provide moment-to-moment data on how environmental factors influence mood and stress, as well as how these relationships are impacted by social interaction, to provide a thorough understanding of the dynamic processes through which environmental exposures may drive mood changes. Important studies such as this will unveil exciting perspectives on the fine-scale mechanisms at play and will fill gaps in the literature, which has previously focused on infrequent measurement of mental health outcomes (eg, every 2 years) or residence-based exposure assessment.

In addition to these high-resolution measures of mobility and psychological processes, we now have access to spatial dataset that provides information on the environment in ways never before seen. Ubiquitous georeferenced street-level imagery, such as Google Street View, provides detailed, time-varying information on specific small-scale environmental factors.^6 7^ Recent advances in deep learning have made it possible for researchers to rigorously and systematically evaluate these images for exposure assessment at scale.^8^ We can now tease out exactly what is in each image, such as sidewalk availability or tree species, and link these images to the locations that they were gathered. These images have also been gathered for over a decade, so that we can evaluate how environments change over time. As mentioned above, measuring specific, time-varying environmental features has been challenging, and has hindered the ability of previous studies to isolate key health-promoting features of the environment. Applying deep learning to street-level images empowers the measurement of environmental factors in a high-resolution, specific, consistent and scalable manner across large areas. Linking these measures to health will reveal policy-relevant and actionable information on how to optimise environments for mental health and well-being

## Modelling policy impacts

Ultimately, the goal of research on the environmental drivers of healthy ageing is to identify potential interventions and estimate how these interventions influence health outcomes. To this end, Yang *et al* employed an agent-based model to evaluate the impact of a free bus policy on both public transit use, as well as depression among older adults. They benchmarked this model against empirical data from England and ran several simulations to examine different policy scenarios. The authors’ model predicted that free bus policies lead to increased bus usage and decreased depression. In addition, improving attitudes towards the bus could enhance the effects of a free bus policy, particularly for those living close to public transit, as well as in scenarios where poorer populations live close to the city centre. Although these agent-based models contain substantial assumptions, they provide crucial information to decision makers to enact policies that maximise health. Agent-based models also highlight the factors that may modulate the effectiveness of environmental interventions, which may indicate the need for multiscale interventions for optimal outcomes.

## Commentary on the MINDMAP Project

With all of the effort that went into harmonising exposure, outcomes and other core measures across six cohorts spanning seven countries (Wey *et al*), the findings gathered in this special issue provide novel cross-national findings. The MINDMAP collaboration has laid a groundwork for future research to harmonise environmental exposure data and health outcome information in multiple large studies across countries in Europe. The initial offering from the MINDMAP Project is only the beginning. Perhaps the best is yet to come.
